# Physicians’ understandings and experience of advance care planning in Norwegian nursing homes: a qualitative study

**DOI:** 10.1186/s12904-024-01481-9

**Published:** 2024-06-24

**Authors:** Liv Ødbehr, Reidun Hov, Harald Sanaker, Åsa Serholt Jensen, Aina Korup, Tuva Sandsdalen

**Affiliations:** 1 Department of Health and Nursing Sciences, Faculty of Social and Health Sciences, Inland Norway University of Applied Sciences, Elverum, Norway; 2 Development Center for Nursing Homes and Home Services, Hamar Municipality, Hamar, Norway; 3 Municipal Medical Center, Ringsaker Municipality, Brumunddal, Norway

**Keywords:** Advance care planning, End-of-life care, Nursing home, Palliative treatments, Palliative care, Physicians

## Abstract

**Background:**

Advance care planning is a way of facilitating conversations with patients about future health care, values, and preferences at end of life. Nursing home physicians have the medical responsibility and the main obligation to facilitate planned meetings with patients. Although there has been a great deal of focus on establishing advance care planning in Norwegian nursing homes, it has yet to be widely implemented. Stated reasons are that the work routines in a nursing home do not include such meetings or that implementation seems complex due to frail patients. The aim of this study is thus to explore how physicians understand and experience advance care planning and follow-up of care plans in Norwegian nursing homes.

**Methods:**

The study has a qualitative research design with a phenomenological-hermeneutic approach based on interviews of twelve nursing home physicians working in community care. Interviews were conducted in February 2023 to May 2023, using a semi-structured interview guide. All interviews were recorded on audio files, transcribed, and analyzed using structural text analysis.

**Results:**

The findings are presented based on the following themes: (1) advance care planning is a dialog and a process, (2) advance care planning implies clarifying mutual expectations, and (3) advance care planning that brings relief and hope to patients is a medical art.

**Conclusions:**

Advance care planning is a complex and dynamic process that implies medical treatment, decisions on treatment level, pain relief, and formulation of care plans where the patient’s self-determination and personal values are respected. It implies an ongoing dialogue between physicians, patients, and their relatives about values such as dignity, self-understanding, social relations, and existential questions at end of life. Advance care planning requires a holistic approach that meets patients’ psychological and existential needs such as comfort, trust, hope, and respect as well as their preferences and concerns.

**Supplementary Information:**

The online version contains supplementary material available at 10.1186/s12904-024-01481-9.

## Background

Physicians in nursing homes have the principal responsibility for the medical aspects of palliative care such as initiating and withdrawing medical and drug treatments, decision-making processes that deal with end-of-life care, as well as ethical and legal issues related to the care of patients in nursing homes (NHs) [1, 2]. Palliative care is an approach to improve the quality of life of patients and their relatives who are facing challenges associated with life-threatening illness [3], including end-of- life care that comprises comprehensive care for persons when it is likely that the remaining life span is significantly limited, often defined as weeks or days. The goal is to prevent or reduce sufferings of any kind whether physical, psychological, social or spiritual [3]. Statistics show that most patients in Norwegian NHs are elderly, with a figure of approximately 39,000, and that nearly half of all deaths among the elderly in Norway each year occur in NHs [4–6]. The proportion of older people above 70 years of age will increase dramatically in Norwegian society towards 2060 [4] and the trend of an aging population is recognized in almost all countries in the world [7–10]. Many patients in NHs have reached an advanced age and are frail due to mental or physical limitations. It is acknowledged that comorbidity intensifies with increasing age and patients in NHs often suffer from one or more chronic diseases which make them more vulnerable [5, 11]. Elderly people with multiple comorbidities often struggle with fatigue and most of the patients in Norwegian NHs need advanced and complex assistance as they receive care around the clock [1, 12, 13]. A typical feature of many patients in NHs is that they lose their capacity to make decisions related to end-of-life care, as around 80% suffer from dementia. This means that both somatic, functional, mental and neuropsychiatric needs must be met.

For treatment and care in the end -of-life to be of high quality, it must thus be well planned [5]. Advance care planning (ACP) is an essential way to facilitate communication about end-of-life care in the NH care context [14, 15]. ACP means planning and preparation of both medical treatment and overall care in the end-of- life [16, 17]. It is an ongoing process of communication including clarification about pain relief, treatment alternatives and treatment level, as well as patients’ goals, values, and concerns at end- of -life [18, 19]. ACP meetings provide an opportunity to talk about the patient’s present and future preferences and include clarifications as to whether patients should be treated in hospital or not if their condition deteriorates. Furthermore, the dialogue must be adapted to the patient’s individual requirements, such as age, cognitive function, disease, experience, and cultural and language background with an anticipation that patients should be offered ACP conversations although the development of the disease makes them more debilitated [19–21]. Such clarifications are particularly important in advance, especially when patients reach the phase where they cannot take care of themselves or are unable to continue such conversations [20, 22, 23].

In this article, we build our understanding of ACP on the definition developed by the European Association for Palliative Care (EAPC), as described above [17]. In addition, we have included the follow-up of ACP in terms of palliative care plans and decisions from ACP meetings in our understanding [12, 24]. The interaction between different professional groups aims to safeguard the quality of care and quality of life for end-of-life patients [20, 25]. The implementation of palliative care plans for each patient in NHs thus involves a team approach. Team members may be e.g., physicians, nurses, other healthcare workers, physiotherapists, or occupational therapists, further referred to as professional care workers in this article. ACP is therefore characterized by a high degree of cooperation and interaction between professionals, where the patient and in some cases the relatives are included as part of the team [20, 26].

End-of-life care in NHs comprises organizational, professional, ethical, and existential expertise [1]. Despite the development of statutory ethical ACP guidelines in Norwegian NHs, the implementation of ACP has not been systematic [20]. Research reveals few NHs that routinely offer and carry out conversations about end-of-life care with patients, even though it is well known that most patients admitted to an NH end their life there [1]. Challenges reported regarding ACP in NHs show that professional care workers reflect little on the purpose, content, and timing and on how to include frail elderly patients in ACP communication [24]. At the same time, physicians and nurses differ in their approach to ACP conversations based on their professional background and what they can bring into such a conversation [27]. In physicians’ training, palliative care is often taught in hospitals where the patients age differs from those in NH [12]. Organizational factors as lack of suitable spaces and shared decision-making can be a barrier for implementing ACP as well as a lack of understanding of the benefits of ACP in NH [28]. This clearly indicates that we need more research on how to conduct ACP in NHs from the perspective of nursing home physicians (NHPs) as well as more knowledge on what kinds of challenges these physicians face in implementing ACP in this specific care context [27]. The aim of the study is thus to explore how physicians understand and experience ACP and the follow-up of care plans in Norwegian nursing homes.

## Methods

The study has a qualitative research design with a phenomenological-hermeneutic approach based on interviews of NHPs, since our focus was on physicians’ understanding and experience of ACP in NHs [29].

### Setting and recruitment of participants

Norwegian NH are organized in the municipal health service. Nurses and other healthcare professionals are employed in the NHs, while many physicians have a combination position and work partly as NHP and partly as general practitioners. NH departments may be organized as open facilities, while others have closed wards in addition. A few nursing homes have specialized palliative care units or palliative beds [4].

The participants in the study were recruited from the network of NHPs in Eastern Norway, the Centre for the Development of Institutional and Home Care Services in Inland County in line with purposive sampling [30]. Information about the study was sent to the leaders of the NHs where the physicians worked. The information was communicated orally to the physicians at a network meeting and by mail with supplementary information. The inclusion criterion was that the physicians had experience from conducting ACP in the NHs where they worked. Sixteen physicians agreed to participate in the interview survey but four of them had not yet conducted ACP and were therefor not included in the interview study. Twelve physicians from the network were contacted based on the inclusion criterion, all working in different municipalities in Inland Norway. In some of the nursing homes, ACP was implemented in a systematic way or in day-to-day operations, while in other nursing homes it was based on signals from the patients that the healthcare professionals responded to. The characteristics of the participants showed that both male (*n* = 8) and female (*n* = 4) NHPs were included, all between twenty-three and sixty-five years of age. Six of the physicians were Norwegians who had completed their education in Norway, while six had studied medicine in other European countries. Their work experience as physicians varied from six months to twenty-two years. Several had extensive experience from working in hospitals (*n* = 6) and some were specialists (*n* = 8) in e.g. geriatrics, or general medicine. One of the participants had completed a PhD. The size of the nursing homes where the physicians worked varied. The lowest number of patients for which an NHP was responsible was six, while the highest number was eighty. The physicians had different job percentages from a 20% part-time job (*n* = 2) to a full-time position (*n* = 7) in the NHs. There were palliative patients in all NHs, and at some NHs there were also palliative units. The physicians’ experience with ACP varied from one to twenty-two scheduled meetings.

### Data collection

Data were collected between February and May 2023 in face-to-face meetings in the NH meeting room, based on the participants’ preferences. A semi-structured interview guide was used as a support in the interview, with a few simple questions built around the research questions (supplementary file). The interview guide was tested initially in a pilot interview and adjusted by modifying individual words before being used in the main interview study. The pilot interview was included in the study as the informant was one of the twelve physicians in the sample. An associate professor and an NHP in the project conducted the interviews (RH, HS). Examples of questions in the interview guide were:

(i) How do you understand ACP? (ii) What kind of experience do you have with ACP in the nursing home where you work? (iii) What content do you emphasize in an ACP meeting? Describe an ACP meeting that you felt was good and one that you felt did not go well.

The interview guide also included questions about quality in care and interdisciplinary collaboration. The interviews lasted from 30 to 60 min and were audio recorded. The recordings were transcribed verbatim and subsequently processed.

### Data analysis

The advantage of phenomenological-hermeneutic interpretation is the dialectic movement between understanding and explanation. In hermeneutics, understanding of a phenomenon is regarded as a continuously evolving process back and force from understanding of parts to a comprehensive understanding. In phenomenological thinking, it is about investigating the meaning of the experiences of the phenomenon being investigated. The combination of these approaches provides rich descriptions of what is to be examined [29].

The analysis consists of four steps: (i) naïve reading, (ii) structural analysis, (iii) comprehensive understanding (interpreting the whole), and (iv) formulating a phenomenological-hermeneutic expression of lived experience [29].

Naïve reading means to let the text speak, enabling the reader to switch from a natural attitude to a phenomenological attitude. The interviews were read through several times by the project group to gain an idea of the meaning of the entire text. The naïve understanding was formulated as a short paragraph and was validated through the structural analysis [29].

Structural analysis is a methodology of interpretation of the text, seeking and identifying themes that convey an essential meaning of lived experience. The text was first divided into smaller meaning-bearing units (meaning units) in the form of sentences that conveyed one meaning. The meaning units were read and reflected on against the naïve understanding and then condensed. Condensation of the text means to keep the essential meaning of parts of the text and describe it in everyday language. The condensed meaning units were then read through and reflected on to find similarities and differences in order to formulate sub-themes. These are grouped into themes and then to main themes. The themes are condensed descriptions containing an elicited meaning. By decontextualizing the meaning units from the whole text, the text- parts were considered independently from their context. The themes where then validated against the naïve understanding [29]. To ensure credibility, all researchers in the research group were involved in the analysis of data (LSØ, RH, HS, ÅS, AK, TS). The members of the research group were in continuous dialogue to reach consensus on the content and labelling of the themes, aiming to ensure that these were in accordance with the content of the interviews.

Themes, sub-themes were summarized in relation to the research question and the context of the study [29]. The comprehensive understanding and a phenomenological-hermeneutic expression of lived experience was then formulated, being aware of our pre-understanding; this was as close to lived experience as possible using everyday language, as presented initially in the discussion of this article. The findings from the analysis were presented at a network meeting for the physicians, followed by a conversation about the content afterwards (Table 1).

**Table 1:** Content analysis

Meaning unit	Condensation	Sub-themes	Themes	Main themes
do not bring up the most difficult topics in the first conversation but in the second or third conversation	address difficult topics gradually	ACP is many dialogs in one process	process of several conversations/ dialogs	Advanced care planning is a dialog and a process
identify expectations in relation to the patient’s situation and further medical plans	expectations and plans for the situations	clarify expectations and make plans	expectations for care plans	Advanced care planning implies clarifying mutual expectations

### Findings

All the informants were physicians by profession. In the presentation of the findings, the terms informant and physician are used interchangeably. The concept of experience includes the physicians’ lived experience based on the phenomenon of ACP, and their reflections on their own experience and that of patients and relatives. Understanding ACP implies a partial understanding and a general insight into how, where in the NH, and when ACP should be offered to patients and their family members.

## Naïve understanding

A clear understanding was that the physicians had a holistic perspective on the patient’s situation. Palliative medical treatment was discussed with the patients, and the physicians emphasized the importance of relating to patients with an attitude of respect and consideration. Palliative treatments were discussed openly and honestly with the patients and their relatives in the ACP meetings. The physicians regarded the dialogue with the patient and family as an ongoing process over time. Not all topics could be addressed at once since many questions were too difficult to decide on immediately. Several conversations during the final phase of life were necessary for most patients.

### Structural analysis

The findings from the structural analysis of the interviews are presented based on the following themes and headings: (1) advance care planning is a dialog and a process, (2) advance care planning implies clarifying mutual expectations, and (3) advance care planning that brings relief and hope to patients is a medical art.

### Advance care planning is a dialog and a process

The interviews revealed that the informants wanted to get to know the patients and their relatives before implementing ACP. Some of the informants believed that ACP should be carried out in the second or third conversation with the patient, as they often had weekly meetings with patients and their relatives. The first ACP meeting was implemented after three weeks of the patient’s stay in the NH. The physicians could then observe the patient for a while with the aim of adapting treatment and care plans to the individual patient. The mutual goals of treatment and care were clarified in these conversations. One of the informants put it this way:

For me, it is important to get to know the patient’s wishes, history, and work experience. Then I know what they have done before in life. Sometimes it feels as if they have appeared in the nursing home “out of the blue” and they have not had a life before. But I find information about their life story during the conversations. (informant 5)

The physicians emphasized that they could seldom talk to the patient about everything related to the end of life in the first meeting. They had to divide the conversation into smaller parts to enable the patient to process questions and answers gradually. In such conversations, decisions about palliative treatment and care were made with the patient. One way of doing that was to start the meeting by listening to the patient’s most important topic first. The physicians emphasized that this would ensure the patient’s self-determination. This also implied that assessing the patient’s competence to consent was carefully clarified by the physicians as early as possible. If a patient lacked the capacity to consent due to dementia, the patient’s close relatives had to respond on behalf of the patient. The physicians agreed that the core question in any ACP with the patient was “what is important to you”. One informant stated:

Advance care planning is not just a conversation, but a dialogue about future treatment and wishes, about how they want things to be and their preferences in the last period of their lives, and what is important for the patients in that phase. That’s how I understand it. (informant 1)

The physicians emphasized the importance of being realistic towards the values of patients and their relatives. They found that it was also common for patients to change their opinion during the palliative process and timeline. Patients often have anxious thoughts during this period, and heavy emotional burdens that they need to talk about. These feelings and thoughts could influence their decisions. Many patients stated that they were afraid of losing autonomy in their lives and wanted to be able make informed choices if possible. Physicians emphasized that it was crucial to show respect and openness towards changes in the perspectives of patients and family members.

### Advance care planning implies clarifying mutual expectations

The physicians considered that mutual openness and clarification of expectations about medical treatment were essential for a good collaborative process. One of the informants had learned that it was always wise to discuss the patient’s nausea and pain before the ACP meeting started so that the patient could relax and fully participate in the dialogue. Furthermore, talking about expectations and possibilities regarding medical treatment was important for both parts. The starting point for the conversation was most often the patient’s concerns and questions. One of the informants said: “If the focus becomes more on what is important for the patient, then we can talk more about that, and less about treatment limitations. Then it’s a good conversation, I think”. (informant 5)

The conversation should also inform the patient about what is meant by palliative treatment and care. One informant said:

In the planned meeting with a patient, I try to support the individual patient to express their needs and ambitions and wishes as far as possible. I ask if they have any thoughts about the future. Patients wonder what they can and cannot do. Then I talk about treatment and illness. It may be how the disease affects them, and how they want things to be in the last part of their lives. (informant 6)

The physicians tried to provide customized information about illness, treatment, and relief to patients and their relatives. Several of the informants said that they also referred to current research in the area and prognoses when talking to patients. One informant put it this way:

In the ACP meeting, I try to get information from patients about what expectations they have in relation to their stay. I also try to provide good information about prognoses from a statistical point of view, and what to expect and to what extent. So I always ask if they are wondering about anything. I also try to explain how the system works, how we work in the department, and on which days there is a planned visit by physicians. (informant 8)

Misunderstandings and disagreements between professional care workers and patients could sometimes hinder good communication and care. Occasionally the physicians found that tensions and conflicts could arise and they therefore understood that it was important to clarify patients’ ambiguities during the process. The physicians emphasized that not every wish from the patient could be followed up. One informant (informant 4) emphasized the challenges involved in disagreements about medical issues. The physicians felt that it was important to listen to the patients’ suggestions but at the same time avoid *medical overtreatment.* By overtreatment they meant that curative treatment was maintained for too long, which was not helpful for patients. Poor cooperation and communication and small conflicts could occasionally arise between the professional care workers, patients or relatives. That could lead to divergent views on the treatment and the palliative care plan. One informant said: “There may be disagreement about treatment. So when we have very sick patients in the long-term ward, and when the family has unrealistic thoughts about how well things may go, then there can be some discrepancies.” (informant 11).

Several of the informants believed that quality in ACP was linked to professional care workers’ knowledge and ability to collaborate within the team and with the physician. The physicians described good collaboration as planned, but still open and flexible where something new could be created in the meeting with the patient. The guidelines for palliative care could easily constrain spontaneous ideas in the interaction they had with the patient. An important question that the physicians found that professional care workers and patients’ relatives often raised was assessments of cardiopulmonary resuscitation. The physicians said that they listened to the opinions of nurses, patients, and relatives on the issue before taking any decision. In these assessments, the physicians also informed patients and relatives about how end-of-life care and the death process could be adapted to them in their situation. They also understood that it was important to explain the health care system to the patient and how the health services worked. The physicians found that it was often useful to regularly summarize for patients how their palliative treatment and care were developing.

### Advance care planning that brings relief and hope to the patient is a medical art

ACP that enabled patients to experience relief and comfort was described by the informants as a *medical art.* In such situations they perceived that the patient and their relatives felt comfortable and well taken care of. When the conversation became too predetermined in the sense that the physician’s need for clarifications came first, they felt that patients easily could perceive the conversation as artificial or superficial. Another obstacle to good planning was poor preparation due to the physician’s heavy workload. The physicians learned that when they managed to understand what patients thought about themselves and their situation, it was easier to meet their existential needs in the conversation. They emphasized the importance of clarifying patients’ wishes for end of life with their relatives. They understood that there had to be an open dialogue throughout. One of the informants said:

Find out what they think about and talk about the end. I ask if they have a faith or belief in God - if they are afraid of something. Many patients have a cognitive impairment before they arrive. A family member then speaks for the patient. I then have separate conversations with the family in those cases where it is important, and then try to use the patient’s previous resources and coping capacity (informant 12).

The physicians felt that the most important part of palliative treatment and care was that the patient perceived good quality of life with less pain and anxiety and a feeling of wellness in spite of their situation. They saw that patients’ feeling of a lack of independence was difficult to handle. One of the informants said that it was “important not to underestimate patients. Many patients are very robust and can tolerate a lot of information about their disease” (informant 9). Here they meant that many patients can bear hearing that they will die from their disease. Problems could sometimes arise when the patients’ relatives were in a grieving process and found it hard to talk about end- of-life topics. Nevertheless, some patients refused to receive information about certain topics that were relevant to their case. In such situations, the physicians felt that it was wrong to force the information on the patient. Profound conversations about existential questions often occurred when conducted at a suitable time. Conversations about death therefore had to be carried out when the patient was ready for it. One informant said: “But there is such an incredibly big difference between a patient with whom you can sit and have a more existential conversation, and one who has no capacity for anything else than saying ‘I want to get better, I just want to get better’”. (informant 2). The informants felt that they mastered this situation by finding the right time and the right place and by being able to understand patients during their suffering.

The physicians pointed out that there is often a dilemma between realism and maintaining hope in end-of-life questions. Finding the balance between realism and ensuring hope was an issue they reflected on. Hope was understood as more than just getting better. Some physicians were afraid of not going deep enough in their conversations with patients. They valued honesty but did not want to be perceived as confrontational or superficial by patients. They felt that an important aspect was to preserve hope and provide reassurance by showing respect and being truthful. The physicians generally did not think that honest talk about the end of life led to patients losing their hope. It was better to talk about things even if they were difficult. They felt that it was always wrong to lie to patients. One informant explained it in this way:

Yes, that’s how hope changes, all depending on your point of view. Even if you know that you will not be alive in three months, you can hope to have the best possible *here and now*, or to reconcile with your family. So that you can have the best possible situation you are in. So it is rather the opposite, giving information about bad news is not something that necessarily takes away their hope. (informant 7)

One of the informants felt that most of the patients appreciated honesty and authentic talk about their serious diagnosis even though it hurt, by saying:

Although the patients might say it from time to time. There are some who may say: “You mean I’ll just die…” But if you’re there and wait, then the conversation will start again, or you can take it up again later. Then the whole thing is reassessed by the patient, and they can hope for the right things, or for what is realistic. (informant 7)

Patients would then feel safer and more secure and most of them understood the situation well, according to informant 3. The patient’s hope was rarely threatened, it just shifted and changed its content.

The ***comprehensive understanding*** of ACP reveals that it is both a dialogue and a process involving several meetings where physicians and patients clarify medical treatments, pain relief, patients’ prospects, and their values. ACP is a holistic approach to end-of-life care where both bio-psycho-social and existential needs are discussed and met. It is also a conversation that includes formulation and reformulations of palliative care plans. ACP implies an honest dialogue and shared understandings between patients, their relatives and physicians. The process is dynamic and exceptionally beneficial, ensuring patients’ self-understanding, explanations, and need for information.

## Discussion

The aim of the study was to explore how physicians understand and experience ACP and the follow-up of care plans in Norwegian NHs. The study’s main findings can be described in a formulation of a phenomenological-hermeneutic expression of lived experience, where the physicians understood ACP as an essential call to take account of patients’ preferences and establish a safe path for the exchange of information between patient, relatives and physicians at the end of life. The ACP included several important clarifications about patients’ possibilities, values and prospects for their future life and upcoming death. ACP was understood as a dynamic process, aimed to meet the patient’s needs for self-understanding, trust, relief, and hope with an attitude of honesty and respect. The physicians found ACP to be a way to follow up care plans including assessments of treatment level. The details of existential questions that deal with life and death could be discussed and reflected on with openness and honesty, as could patients’ anxieties and worries. The physicians described this as a medical art when they saw that the patients’ existential needs were met. We discuss these findings in the following.

## What is known before, and what does this study add?

The informants in this study emphasized that ACP means a dialogue and a process over time. The informants’ understandings were fully in line with international research and consensus on the content of ACP [16]. ACP provides a framework for the actual conversation between physicians and patients, where they can clarify ongoing medical treatments and expectations regarding end-of-life care in a way which strengthens personal dignity to enable patients to live their final days in line with their values. ACP is thus a suitable setting for repeated conversations, because not all elucidations can be made at once [16]. Various topics are addressed, and the conversation includes a presentation of opportunities and limitations for the patient’s path forward with their illness. The basic idea is that many patients experience a form of grief at the end of life, which is a demanding process that touches on existential themes such as hope and meaning, the desire to live on, accompanied by grief and realism. Here, the physicians in the current study elaborated this more clearly than the EAPC definition of ACP by claiming that several ACP meetings, not merely one, are needed with each patient, as patients often change their preferences as the disease progresses. Internationally, there is still ambiguity in the understanding of the term ACP [17] and the content of ACP varies substantially [16, 19]. The physicians in this study seemed to have some form of consensus in their understandings of ACP for elderly people in the NH context.

Guidelines for topics and relevant questions that could be asked in ACP meetings have been developed to help physicians in their work [17, 20, 22, 24]. The physicians in this study emphasized the significance of guidelines in their practice [16], yet they found that the guidelines occasionally could be an obstacle to an open, flexible, and spontaneous dialogue with patients. Although there has been considerable focus on the establishment of good guidelines for palliative care and routines for implementing ACP in eldercare, research shows that there are some barriers in practice. Stated reasons are negative and unclear perceptions of the significance of ACP from health care professionals [31, 32]. A recent scoping review reveals that lack of knowledge of the content of ACP is a key contributing factor to non-implementation of ACP in practice [31]. The review also points out that patients express a fear of losing independence and autonomy through such conversations, and that they find the focus on end-of-life care to be depressing, stressful, and emotionally demanding [31]. These findings strengthen the relevance of our study and show that there is a need for research that emphasizes how ACP can be implemented, especially in the early disease phase while patients are still able to participate in such conversations. ACP meetings aim to enable patients to summarize their own lives, strengthen some relationships and end others, and prepare for their final days. The EAPC consensus on ACP highlights a need for guidance on the timing of ACP meetings, i.e., not too early in the process but not too late, such as shortly before death [17]. Likewise, there is a need for more research on how guidelines for ACP can be designed to safeguard the structure and content as well as the spontaneity. It seems that the EAPC also confirms this by shifting its focus from instructions for healthcare professionals to the process of communication across ages and illnesses [17].

To work in an NH requires a general level of competence in providing basic palliative care. Competence in medicine is described as a holistic combination of knowledge, attitudes, skills, and values in specific activities [33]. The physicians in the current study focused on factors beyond medical treatment. In some descriptions of relevant competence in ACP, decision- making and networking are mentioned [31]. Research also reports a lack of systematic clinical routines to support the implementation of ACP discussions in practice. That implies that decisions are not followed up by interprofessional teams [26]. This was also a point the physicians emphasized in this study by highlighting the importance of the interdisciplinary team’s efforts to help the patient. It is crucial to follow up the palliative care plan; the plan itself is not enough. An interdisciplinary team ensures the follow-up, changes, and reassessments along the way. The physicians are dependent on the information from the interdisciplinary team. In this study, the physicians stated that the team is no stronger than its weakest link, by which they meant that the competence of everyone in the team played a role.

It was clear that NHPs in the current study found ACP to be a comprehensive approach to patients in their palliative phase and they saw medical treatment as only one aspect of overall care. The starting point for ACP is a holistic perspective on the patient’s everyday situation, including the physical, psychological, social, spiritual, and existential needs [11]. The physicians in the current study evaluated their presence in the dialogue with patients and emphasized principles such as trust, honesty, openness, and respect. In such meetings, something new was created in the relationship and in the mutual understanding. This is much in line with the consensus of the content of ACP, where needs like comfort, independence, and dignity are particularly mentioned and should be included [16]. A Norwegian study of newly qualified physicians found that qualities such as calmness and acceptance were seen as more appropriate than heroic action when death was imminent [34]. The physicians in this study shifted their focus from treatment to symptom relief and adjusted their words and decisions to the patients’ life story. During the learning period they tried to balance uncertainty and ambiguity while searching for ways to assist and guide patients [12]. The study confirmed some of the aspects that the physicians in this study also emphasized, i.e., they learned by doing and learned the medical art of ACP by practicing it.

There are high demands and great responsibility on the shoulders of NHPs in NHs, yet relatively few research studies describe how NHPs explain strengths and limitations in the work of supporting grieving patients in end-of-life care [34]. In NHs, a large proportion of patients have dementia, placing especially high demands on physicians and other health personnel and raising the need for a person-centred care approach [35, 36]. Each patient is unique and must be met on the basis of their particular situation and history. Studies show that patients often need support from family members to express requests in the decision-making process [16]. Generally, the physicians had a positive view of the meetings with patients and their relatives, but a few times conflicts or tensions could arise. The NHPs underlined that ethical questions had to be asked and that they needed to convey awareness of the mourning process in the patients’ life. Sometimes the physicians felt the burden of the responsibility, especially in cases of disagreement on the content of the medical treatment plan. A study by Thoresen et al. [27] emphasizes that dying at an old age is described by many as a natural part of life. Nevertheless, such experiences can be complex and disruptive and vary greatly between people. Most patients emphasize that they want to die with their identity and self-image intact. Nevertheless, studies indicate that patients’ autonomy and legal rights are often neglected or scarcely taken into account [27]. The physicians in the current study acknowledged that this was sometimes demanding in situations where the patient suffered from dementia. In a recent study by Jansen [34], it emerged that NHPs experienced existential vulnerability as a burden of powerlessness and guilt when faced with difficult treatment compromises in relation to patients. Existential vulnerability is seen as the vulnerability of being reminded of one’s own death [34]. The physicians in this study did not express it in such strong terms, but a few of them touched upon similar notions. In contrast, the physicians said that they were very brave in initiating honest face-to-face conversations with patients and relatives about end-of life care. One reason might be that many of the physicians we interviewed had worked for a long time in the nursing home and had extensive experience as physicians. In summary, it appears that there is a need for better organization of the services, as well as systems and routines that facilitate the implementation of ACP in NH. Shared knowledge and better collaboration between healthcare professionals might break down barriers for ACP in practice and increase quality in NHs with less experience of ACP.

### Strength and limitations

A weakness of the study might be that we included participants who are engaged and interested in ACP. This may have influenced our findings by mainly reflecting positive experiences of ACP.

Dependability was ensured in that the selection of informants included wide variation in experience, age, gender, and nationality. Equivalence and internal consistency were maintained by the same two members of the research group (a physician and a nurse) conducting the interviews (RH, HS) and ensuring good follow-up questions; this also reduced the possibility that their preconceptions had a strong influence. Physicians working in practice may sometimes be difficult to recruit. We considered twelve participants to be satisfactory. A semi-structured interview guide was used during the interviews. The guide was piloted, adjusted, and implemented after some small modifications of words. The interviews were rich and provided a wealth of transcribed text, and the pilot interview was thus included in the data. Joint reflection in the research group on the four steps in the analysis helped to develop the content in the findings. Every step during data collection and data analysis was documented, such as the use of quotes and examples in the results section of the article (LSØ).

A few similar studies have been conducted in NHs with interviews of physicians, and these strengthen the validity of the current study [6, 11, 13, 27]. To enhance the transferability of findings, we have searched for reflective journals and detailed descriptions in other international and Nordic studies. However, qualitative data depend on the context and are therefore not directly transferable. Overall, a potential weakness and strength in this study is that all research group members had a healthcare background and clinical experience. Some had experience from nursing homes, and all had experience with patients in the palliative phase. The researchers’ experience and background may also have been a key factor in the analysis and interpretation of data. The research group therefore worked back and forth with the textual material to bring out the informants’ voices. The steps and findings in the analysis were discussed until agreement was reached on the formulation of the themes.

## Conclusions

Advanced care planning is a complex and dynamic process that implies medical treatment, decisions on treatment level, pain relief, and formulation of care plans where the patient’s self-determination and personal values are respected. It implies an ongoing dialogue between physicians, patients and relatives about values such as dignity, self-understanding, social relations, and existential questions at the end of life. Advanced care planning requires a holistic approach that meets patients’ psychological and existential needs such as comfort, trust, hope, and respect as well as their preferences and concerns.

### Electronic supplementary material

Below is the link to the electronic supplementary material.

Supplementary Material 1

Supplementary Material 2

## Data Availability

The datasets used and/or analysed during the current study are available from the corresponding author upon reasonable request.

## Abbreviations

ACP: Advance care planning
NH: Nursing home
NHP: Nursing home physician
GDPR: General Data protection Regulation
