# Supplementary material for: Physicians’ understandings and experience of advance care planning in Norwegian nursing homes: a qualitative study
Source: BMC Palliat Care. 2024 Jun 24;23:158. doi: 10.1186/s12904-024-01481-9 (PMC11194902; doi:10.1186/s12904-024-01481-9)
Supplement: Supplementary file 1 — Supplementary Material 1 [file 12904_2024_1481_MOESM1_ESM.docx]

**Supplementary file 1. Interview guide.**

**Content of the interview guide for the study: Quality of palliative care and experiences of advance care planning in Norwegian nursing homes; patients and physicians’ views (PalCare).**

What characterises (good) palliative care ?

What is your experience in providing palliative care to life - threatening ill and dying patients in nursing homes?

- Can you please tell about a situation where the care was good?

Which factors promoted the possibility to provide good palliative care?

- Can you please tell about a situation where the care was not so good?

Which factors inhibited the possibility to provide good palliative?

Please describe how you assess the importance of advance care planning for the quality of palliative care in the nursing home.

- What is your experience with advance care planning (ACP-meetings) in the nursing home where you work
- How do you assess the need for follow-up calls after ACP meetings

Follow-up questions were used, such as: Can you please explain more about that…?
